# Improving Lexicosemantic Impairments in Post-Stroke Aphasia Using rTMS Targeting the Right Anterior Temporal Lobe

**DOI:** 10.3390/brainsci16010117

**Published:** 2026-01-22

**Authors:** Sophie Arheix-Parras, Sophia R. Moore, Rutvik H. Desai

**Affiliations:** 1 Department of Psychology, University of South Carolina, Columbia, SC 29208, USA; srm21@email.sc.edu (S.R.M.); rutvik@sc.edu (R.H.D.); 2 Institute for Mind and Brain, College of Arts and Sciences, University of South Carolina, Columbia, SC 29201, USA

**Keywords:** rTMS, aphasia, stroke, lexicosemantic

## Abstract

**Background/Objectives**: Repetitive Transcranial Magnetic Stimulation (rTMS) can enhance post-stroke aphasia recovery. The right Inferior Frontal Gyrus is the most common target in rTMS studies for inhibitory stimulation. However, lexicosemantic processes involve a large brain network including the Anterior Temporal Lobe (ATL). We hypothesize that rTMS targeting the ATL will improve lexicosemantic impairments in people with post-stroke aphasia. **Methods**: In a Single-Case Experimental Design, three people with post-stroke aphasia with lexicosemantic impairments performed Picture and Auditory Naming and Semantic Decision tasks five times a week for one or two weeks to establish baseline scores. Then, each participant received continuous inhibitory Theta Burst Stimulation targeting the right ATL, five times a week for two weeks. After each rTMS session, participants performed all linguistic tasks. A follow-up measurement was performed one month after the end of the study. **Results**: All participants showed significant improvement in the Picture Naming task, while only P1 improved in Auditory Naming accuracy. In the Semantic Decision task, only P2 showed improvement in both accuracy and RT, while P1 showed improvement in RT alone and P3 showed no improvement. **Conclusions**: The results suggest that ATL could be a potential target for future brain stimulation studies in aphasia involving lexicosemantic impairments. RTMS targeting the ATL may modulate the connected ventral semantic stream, leading to improvements in lexical access. This preliminary study highlights the possibility of selecting the cortical target for rTMS based on the clinical profile of the participant, an approach that will need further investigation in larger sham-controlled studies.

## 1. Introduction

Between 18 and 66% of stroke survivors suffer from aphasia in the United States of America [1]. Aphasia significantly restricts participation in social, familial, and professional spheres, profoundly impacting the quality of life [2,3]. While Speech and Language Therapy (SLT) is the gold standard for aphasia recovery, it often does not lead to complete recovery [4].

Non-invasive brain stimulations, such as transcranial Direct Current Stimulation (tDCS) or repetitive Transcranial Magnetic Stimulation (rTMS), are promising tools for enhancing post-stroke aphasia recovery [5,6]. Contrary to tDCS, rTMS alone has proven to be effective for post-stroke aphasia recovery with or without being associated with online language tasks or SLT [5,6]. Majority of rTMS studies in post-stroke aphasia following a left hemisphere stroke applied inhibitory stimulation to the right Inferior Frontal Gyrus (IFG). This approach is based on the theory of transcallosal interhemispheric imbalance, where a brain lesion leads to hyperactivation of the undamaged hemisphere and hypoactivation of the damaged hemisphere [7,8]. However, targeting the right IFG for all individuals with post-stroke aphasia may be inefficient [9]. This method may overlook the complex organization of language, which involves a large, interconnected network, also linked to other cognitive functions such as executive functions and working memory [10]. Stefaniak et al. [9] indicated that people with different aphasia profiles may have varied recovery paths. In an rTMS study targeting the right IFG in people with post-stroke aphasia, Dresang et al. [11] found that semantic and phonological baseline abilities (measured using the Philadelphia Naming Test error types, [12]) could predict rTMS outcomes. More specifically, they observed that higher baseline semantic scores were associated with greater naming improvement in the rTMS group compared to the sham group, whereas individuals with higher phonological scores appeared to show reduced benefit from rTMS [11]. This suggests that a participant’s language profile may influence their response to rTMS. The precise clinical profile of participants can potentially inform the most effective cortical target to facilitate or inhibit. A study highlighted this approach, showing that participants with phonological impairments benefited from inhibitory rTMS targeting the right motor cortex of the lips [13]. As rTMS not only modulates the targeted area but also influences the connected network through intra- and interhemispheric connection, improvements in accuracy and the reduction in phonological errors in a Picture Naming task following motor cortex stimulation may be attributed to the stimulation of the dorsal phonological stream [10,13,14].

For those with lexicosemantic difficulties, post-stroke aphasia can result in anomia and various semantic errors (e.g., saying *wolf* instead of *dog*). These difficulties can result from a deficit in lexicon access or the semantic system itself [15]. Lexicosemantic processes involve a complex brain network including several cortical areas [16]. According to the hub-and-spokes theory, the Anterior Temporal Lobe (ATL) serves as a central amodal semantic hub, integrating information from various modalities [17,18]. Other theories emphasize its multimodal and potentially lexical sensibility [16,19].

Here we targeted, for the first time to the best of our knowledge, the right preserved ATL using inhibitory rTMS in persons with post-stroke aphasia characterized by lexicosemantic difficulties. We hypothesized that participants would improve in naming ability, as well as other lexicosemantic processes such as semantic judgment. We also hypothesized that this approach would improve communication abilities monitored using a self-report questionnaire and a speech production task.

## 2. Materials and Methods

### 2.1. Participants

**Figure 1:**
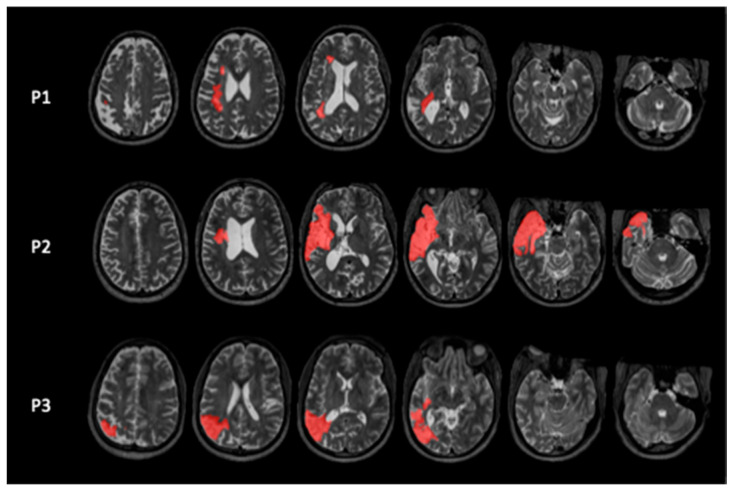
T2-weighted MRI scans showing the location of left hemispheric lesions in red.

We included three participants with chronic mild to moderate chronic aphasia following a left hemispheric stroke (Figure 1) at the University of South Carolina. The study was approved by the Institutional Review Board (IRB) of the University of South Carolina (Pro00138658), and all participants signed an Informed Consent form. They received monetary compensation for their effort.

All participants were right-handed (Edinburgh questionnaire, [20]), fluent native English speakers, had a single stroke event, and were diagnosed with aphasia by a Speech and Language Pathologist or a physician. We excluded participants with contraindications to rTMS such as uncontrolled epilepsy, intracranial ferromagnetic body, or factors that lower seizure threshold, following the safety recommendation of Rossi et al. [21]. Non-inclusion criteria also concerned psychiatric history, illiteracy, known neurodegenerative disorders, or severe uncorrected visual or hearing impairment. None of the participants was receiving concurrent Speech and Language Therapy. Demographic characteristics of the participants are shown in Table 1.

All participants underwent language assessment prior to the experiment conducted by a Speech and Language Pathologist, that included the bedside Western Aphasia Battery (WAB) [22] and the short form of the Philadelphia Naming Test [23] (see Table A1). They all presented with lexicosemantic impairment characterized by anomia and produced semantic errors. They had no major phonological deficit (i.e., preserved word repetition).

P1 presented with moderate aphasia characterized by long latencies and reduced spontaneous speech, primarily due to anomia. Picture and object naming tasks resulted mainly in nonresponses and long delays. Repetition was preserved for both single words and simple sentences, though he could not repeat longer sentences fully. Auditory comprehension was largely intact, although he made occasional errors on complex yes/no questions.

P2 exhibited mild fluent aphasia, with spontaneous speech marked by long latencies, semantic errors, and circumvolutions. Minor sequalae of apraxia of speech were observed such as disrupted prosody and initial phoneme repetition. Despite word-finding difficulties in spontaneous speech, he did not show anomia during the naming tasks. Repetition was preserved for words or short sentences but impaired for longer ones. Auditory comprehension was preserved for short sentences, though difficulties emerged with increased sentence length.

P3 presented mild aphasia with fluent speech, although frequent pauses were noted due to word-finding difficulties. She produced semantic errors both in spontaneous speech and during naming tasks. Repetition was preserved at the word level but showed errors at the sentence level. Auditory comprehension was intact for both simple and complex sentences.

### 2.2. Experimental Design

We used a randomized multiple-baseline design across participants following Single-Case Experimental Design (SCED) protocols [24,25], which is suitable for interventions with potentially long-lasting effects. This design involves intrasubject analysis, repeated measures, and randomization of the baseline’s length.

**Figure 2:**
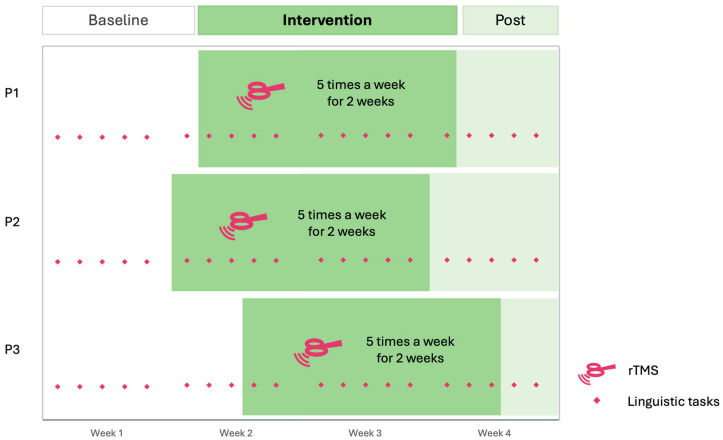
Multiple-baseline design with the baseline duration assigned to each participant. The intervention period is shown in dark green.

Each participant performed three linguistic tasks five times a week over four weeks (Figure 2). These tasks included a Picture Naming task, an Auditory Naming task, and a Semantic Decision task. The length of the baseline phase varied among participants (pseudo-randomized between 5 and 9 baseline points), followed by a two-week period of rTMS administered five times a week (10 sessions in total). The rTMS was used without concurrent SLT and it targeted the right ATL using inhibitory stimulation. Participants underwent a post-intervention period extending to the end of the fourth week of inclusion (1 to 5 follow-up points). This results in 6 baseline points for P1, 5 for P2 and 8 for P3. Each participant completed at least five baseline and five intervention data points. Varying the number of baseline points across participants is a key requirement of SCED methodology and helps ensure that observed changes are not attributable solely to performance variability or repeated task exposure (e.g., systematic improvement after a fixed number of sessions). Additionally, participants completed weekly the Stroke and Aphasia Quality of Life Scale-39 as self-report questionnaires of communication abilities [26], as well as the Peer Conflict Resolution (PCR) task [27], both measures accounted as generalization effects of the stimulation. A follow-up measure was recorded one month later with the same tasks.

### 2.3. Behavioral Measures

For all three linguistic tasks, the order of the trials was randomized and controlled by e-prime software 3.0. Before each task, the participants performed several trials with corrective feedback if necessary to ensure the good comprehension of the instructions.

#### 2.3.1. Naming Tasks

The Naming tasks comprise one Picture Naming and one Auditory Naming task. Before starting the Auditory Naming task, the participants (with the exception of P1) underwent a familiarization phase where all auditory definitions were played, and the corrective feedback was given to the participant orally. As this familiarization phase was not administered for P1, their first session was excluded from the analysis.

The Auditory and Picture Naming tasks included the 36 pictures and 36 definitions from the Auditory and Visual Naming Test [28]. Supplementary items were added to the task. For the Picture Naming task, we added 10 items with late age-of-acquisition words [29]. For the Auditory Naming task, we added 10 abstract items [30] with high frequency [31] (Table A2). Auditory definitions were generated by Chat GPT-4o using a prompt following Hamberger et al.’s guidelines [28] (i.e., “give a short description for this word that can be presentable within 4 s, with low likelihood that the target word can be named before the final word”). All added items (pictures and definition) were tested on a group of 20 neurotypicals (mean age = 60.5, SD = 7.2), and we selected items with a naming agreement >60% (pictures: mean = 80.0%, SD = 15.09; definitions: mean = 88.0%, SD = 9.78). To maximize regularity between sessions, Auditory Naming items were produced using Typecast, an Artificial Intelligence virtual actor service (character casted: Carl, https://typecast.ai).

#### 2.3.2. Semantic Decision Task

These tasks contain 82 stimuli, with half concrete (mean = 4.54; SD = 0.29) and half abstract (mean = 2.51; SD = 0.51) words that were presented to participants on the screen [30]. They were asked to press the “k” key when the word was concrete and “j” if the word was abstract. Response accuracy and reaction time were automatically recorded.

#### 2.3.3. Peer Conflict Resolution

Participants performed the Peer Conflict Resolution (PCR) task [27,32] as a measure of discourse production. First, they were asked to immediately recall a story in their own words, and then to answer questions about the story. To evaluate performance, we analyzed the transcription using the CLAN [33,34] and obtained various measures such as the mean length of utterance, the fluency, and frequency of words. We also assessed the semantic coherence and logical flow with Chat GPT-5, providing it with the PCR task instructions, including the story participants worked on [27]. The model was then prompted with “What is the coherence and logical flow of the following text sequences corresponding to the task provided earlier, on a scale of 0 to 100? Present a score for (1) the retelling part and (2) the questions part”. Semantic coherence scores were compared against those from neurotypical participants with high SES scores from the ABC database [35]. High SES was defined as Hollingshead scores greater than 55 (*n* =10; 7 female; mean age = 67.4 years; SD = 5.0). To rule out potential cognitive impairments, only participants with Montreal Cognitive Assessment scores ≥26 were included [36]. In the neurotypical sample, mean semantic coherence was 67.4 for the retelling part (SD = 7.0) and 74.0 for the questions part (SD = 9.3). To ensure reproducibility of the ratings, we applied the same rating system at two different time points, yielding similar patterns of results. Correspondence between ratings was assessed using Pearson correlations. For P1, correlations were r = 0.83 for retelling and r = 0.74 for question scores; for P2, r = 0.21 and r = 0.47; for P3, r = 0.95 and r = 0.97. Correlations for P2 were lower than those observed for the other participants. To improve transparency, rating justifications generated by ChatGPT-5 are provided in Table A3.

#### 2.3.4. SAQOL-39

The SAQOL-39 is a self-report quality of life questionnaire specifically adapted for people with aphasia [26]. It comprises four subdomains (i.e., physical, psychosocial, communication, and energy) that assess areas of functioning potentially affected by stroke and aphasia.

### 2.4. rTMS

We applied rTMS with a 75 mm figure-8 coil (Cool-B65 Butterfly Coil, MagVenture, Farum, Denmark) connected to a MagPro device. Participants received five sessions per week over two weeks targeting the right ATL using continuous Theta Burst Stimulation (600 pulses, burst frequency at 5 Hz, burst of three pulses at 50 Hz). The stimulation intensity was set at 80–90% of the resting motor threshold (RMT). The RMT was defined as the lowest intensity that elicited a motor evoked response in the relaxed left-hand muscles in 50% of trials. As rTMS targeting the ATL may cause discomfort due to facial nerve twitching, the stimulation intensity was varied from 90% to 80% of the RMT depending on participant comfort, resulting in the following stimulation intensity: P1: 80%, P2: 90%, P3: 80%. The target localization was facilitated by the neuronavigation software Brainsight (version 2.5.5)^®^, which helped to localize the right ATL based on each participant’s anatomical T1 scan. The stimulation target was identified at 10 mm posteriorly from the tip of the middle temporal lobe.

### 2.5. Analysis

#### 2.5.1. Data Analysis and Cleaning

For both Naming tasks, accuracy and reaction time (RT) were analyzed using the speech analysis software CheckVocal (version 3.0.2) [37]. For Picture Naming, RT was calculated starting from the display of each picture. For Auditory Naming, RT was calculated starting from the last phoneme of each description, as the length of each description could vary.

For the Semantic Decision, accuracy and RT were automatically recorded. To clean data, we deleted RTs <200 ms and >3000 ms.

#### 2.5.2. Statistical Analysis

Statistical analysis followed SCED guidelines [38,39]. Initially, we calculated the monotonic trend of the baseline. If the baseline showed a significant trend, we adjusted the results using Kendall’s Tau rank correlation coefficient [40]. This correction involved calculating a baseline-corrected Tau-U if significant positive or negative trends are observed before intervention. This approach allows separation of repetition or learning effects due to repeated exposure to the same stimuli from potential effects associated with the intervention. Specifically, if a learning effect is present during the baseline, it is taken into account when estimating the intervention effect by computing a baseline-corrected Tau. For example, when a positive baseline trend is detected, a significant intervention effect requires the intervention trend to exceed the baseline trend [38,39].

The Tau-U calculation generates effect sizes with the following results: <0.2 corresponding to small effect, 0.2–0.6 moderate effect, 0.6–0.8 large effect and >0.8 very large effect [38,39]. All data visualization aligned with SCED recommendations [25] and were generated using RStudio (4.4.0).

## 3. Results

All participants completed 21 sessions, including the 1-month follow-up assessment. However, P1 rescheduled three consecutive sessions after the seventh session (the first rTMS session) due to being out of town. We also encountered a recording issue with the Semantic Decision task for Participant 3 in Session 9. All sessions were carried out without other interruptions.

### 3.1. Naming Tasks

#### 3.1.1. Picture Naming

**Figure 3:**
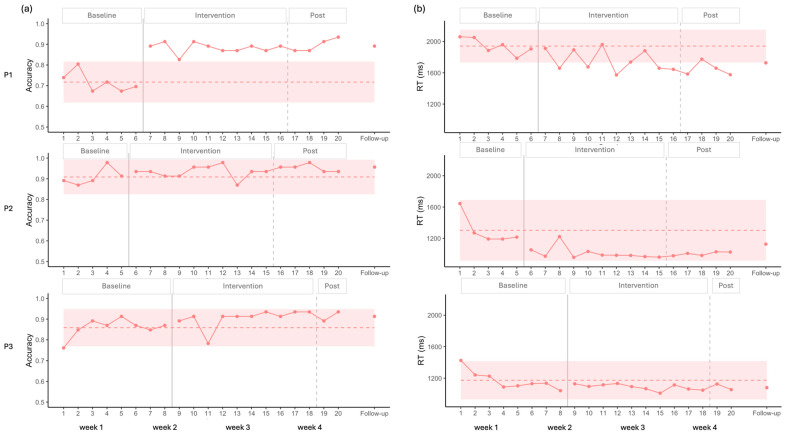
(**a**) Accuracy (**left**) and (**b**) RT (**right**) during the Picture Naming task. The dashed line represents the mean baseline, and the colored envelope indicates +/− 2 SD from the mean baseline.

Visual analysis of the accuracy for P1 showed a stable baseline, followed by a clear increase after the onset of the intervention, with all data points falling above the 2SD band (Figure 3). Statistical analysis supported these findings, indicating no baseline trend (*p* > 0.05), and a large positive effect size (Table 2). This improvement was maintained at follow-up.

For P2, visual analysis indicated a stable baseline, with no observable effect of the stimulation on accuracy. A potential ceiling effect was noted, as most data points were above 90%. Statistical analysis confirmed these results, showing no significant effect of stimulation (*p* > 0.05, Table 2).

In the case of P3, visual analysis revealed no significant baseline trend for accuracy. However, the first baseline data point was lower than the others, resulting in a wider 2SD envelope. Consequently, all intervention points remained within the 2SD band, suggesting no clear visual effect of the intervention. In contrast, statistical analysis indicated a significant moderate effect size (Table 2).

Regarding RT, a negative trend was observed across participants during the initial baseline points; however, statistical analysis of the complete baseline indicated no significant trend (Table 2).

For P1, visual analysis revealed a significant decrease in RT (Figure 3), with eight data points falling below the 2SD band, and a moderate effect size that sustained at follow-up. Statistical analysis supported these findings, showing a moderate decreasing effect of intervention (Table 2).

For P2, visual analysis showed an initial decrease in RT followed by stability, with all points remaining within the 2SD band, suggesting a possible floor effect. Nevertheless, statistical analysis captured the decrease in RT, indicating a significant moderate decreasing effect size (Table 2).

For P3, both visual and statistical analyses indicated no improvements.

#### 3.1.2. Auditory Naming

For accuracy, all participants displayed very low initial and/or second data points compared to the rest of the baseline, suggesting an initial learning effect. Subsequent baseline points were more stable, and statistical analysis did not indicate any significant trend.

**Figure 4:**
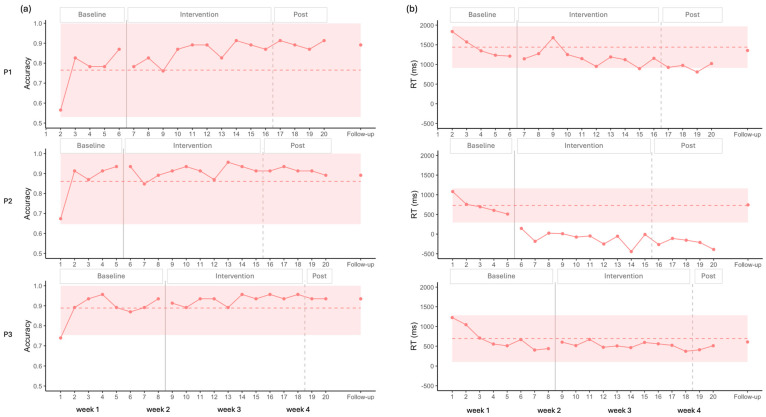
(**a**) Accuracy (**left**) and (**b**) RT (**right**) during the Auditory Naming task. The dashed line represents the mean baseline, and the colored envelope indicates +/− 2 SD from the mean baseline.

For P1, visual analysis showed an increase in accuracy alongside a ceiling effect, as the 2SD band approached the maximum score, leaving little room for improvement. Nevertheless, complementary statistical analysis detected a moderate positive effect size (Figure 4 and Table 2).

P2 and P3 exhibited similar patterns, with no changes induced by stimulation according to both visual and statistical analyses. A ceiling effect was also observed in these cases, with the 2SD band encompassing the maximum value (Figure 4 and Table 2).

A common decrease in RT was observed across all three participants during the baseline, which was statistically significant in each case (P1: Tau = −1.000, *p* = 0.027; P2: Tau = −1.000, *p* = 0.027; P3: Tau = −0.786, *p* = 0.009).

For P1, visual analysis showed two points under the 2SD band in RT, suggesting improvement (Figure 4). However, statistical analysis did not confirm this trend. When accounting for the baseline trend in the Tau calculation, the decrease observed during baseline was no longer present after the onset of the stimulation, resulting in a significant baseline-corrected Tau, indicating a stabilization of performance after the onset of the stimulation (Table 2).

For P2, all intervention points fell below the 2SD band in the visual analysis for RT, again suggesting improvement (Figure 4). However, statistical analysis did not confirm this effect. Moreover, this participant showed negative RT values, reflecting responses given before the end of the auditory description (Table 2).

For P3, visual analysis did not show any improvement for RT (Figure 4). Baseline-corrected statistical analysis confirmed a pattern similar to that of P1, with the baseline trend discontinuing after stimulation (Table 2).

Overall, the results suggest a possible floor effect across participants, which appears more pronounced for P1 and P3 who may have been waiting for the end of the definition before responding (see Section 4.5).

### 3.2. Semantic Decision

**Figure 5:**
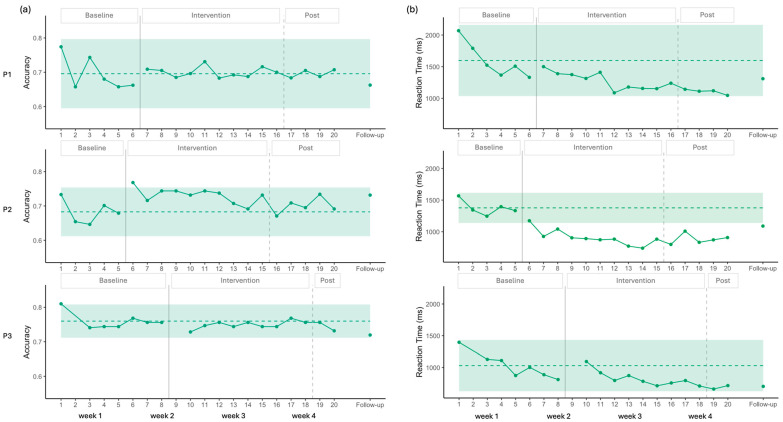
(**a**) Accuracy (**left**) and (**b**) RT (**right**) during the Semantic Decision task. The dashed line represents the mean baseline, and the colored envelope indicates +/− 2 SD from the mean baseline.

Visual analysis showed a stable baseline for P1 and P3 in accuracy. The 2 SD band encompassed all data points following the introduction of the intervention, indicating no change in accuracy (Figure 5). Statistical analysis was consistent with this observation, showing no baseline trend (*p* > 0.05) and no significant change after the intervention was introduced (Table 2).

For P2, the baseline was also stable, and only one data point during the intervention phase exceeded the 2SD band, suggesting a small improvement in accuracy. Statistical analysis confirmed the absence of a baseline trend (*p* > 0.05) and indicated a positive trend with a moderate effect size following the introduction of the intervention (Table 2). However, this improvement was not maintained post-intervention, and no gains were evident at follow-up (Figure 5).

For P1, visual analysis showed no baseline trend and then a decrease in RT; however, all intervention data points remained within the 2 SD band, indicating no meaningful change (Figure 5). Statistical analysis confirmed no baseline trend (*p* > 0.05) and identified a moderate decrease in RT following the introduction of stimulation (Table 2). At follow-up, the improvements observed were not maintained.

For P2, visual analysis showed a clearer improvement in RT. Following the first stimulation session, all intervention data points fell below the 2SD band (Figure 5). Statistical analysis indicated no baseline trend (*p* > 0.05) and demonstrated a large effect size for the intervention (Table 2). At follow-up, RT scores remained below the 2SD envelope, suggesting maintenance of effect.

For P3, visual analysis showed a decreasing trend in RT across the eight-session baseline. This decrease was statistically significant, with a very large effect size (Tau = −0.810, *p* = 0.016). During the intervention phase, all data points remained within the 2SD band. After correcting for the baseline trend, statistical analysis indicated a positive effect size for RT (Table 2), indicating the RT decrease observed during the baseline was no longer present, likely reflecting a floor effect, with the lowest mean reaction time observed at session 19 (661 ms). Thus, the apparent negative effect of the intervention can be explained by the floor level already reached during baseline.

### 3.3. Peer Conflict Resolution

**Figure 6:**
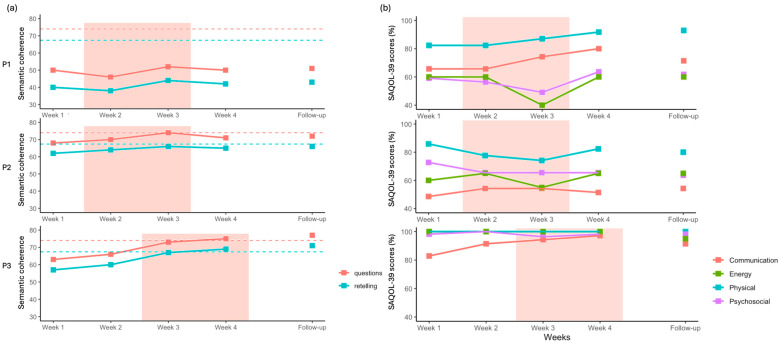
(**a**) Semantic coherence scores (**left**) for the retelling and question answering, with the neurotypical mean shown as a dashed line and (**b**) SAQOL-39 scores in percentage (**right**) for each domain. The shaded orange area indicates the stimulation period (Weeks 2 and 3 for P1 and P2; Weeks 3 and 4 for P3).

For P1, semantic coherence scores for both story retelling and questions answering showed a small increase at week 3 but remained below the neurotypical range (Figure 6). For P2, a slight increase was observed during the two weeks of stimulation; however, this improvement was not fully sustained at week 4 or at follow-up. For P3, semantic coherence scores showed a larger increase after the onset of stimulation at week 3. This improvement was maintained at follow-up, with scores exceeding the neurotypical mean for both measures.

**Figure A1:**
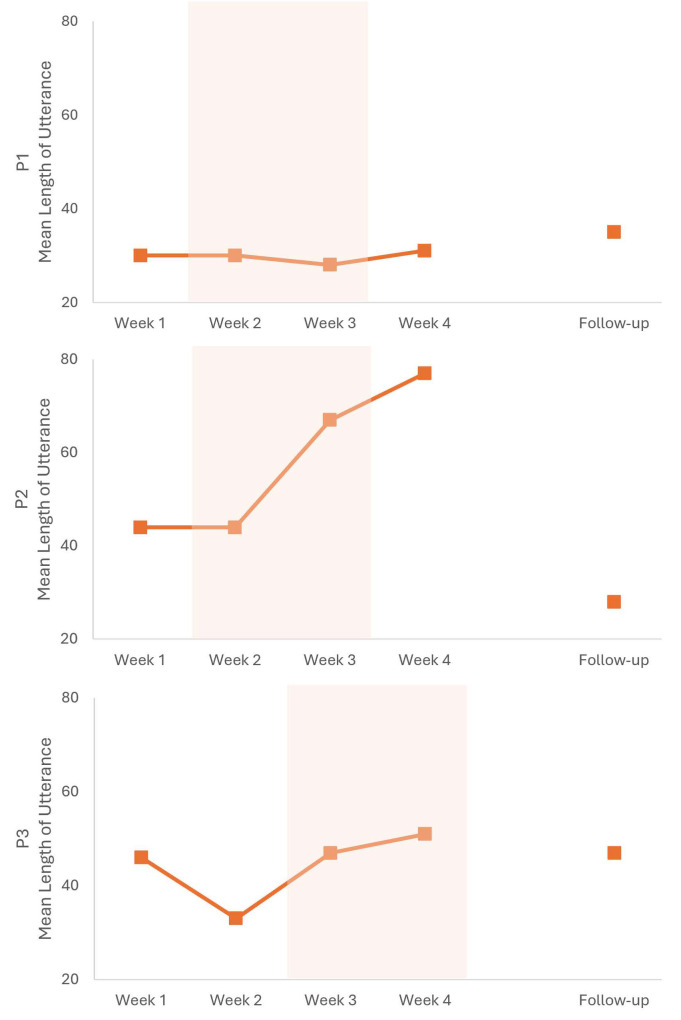
Mean length of utterance (total number of utterances) during the PCR task. Utterances are a string of words that must follow three rules, which is be followed by at least a one-second pause, has proper grammatical structure, or ends with a terminal intonation contour. Shaded orange areas indicate stimulation period.

**Figure A2:**
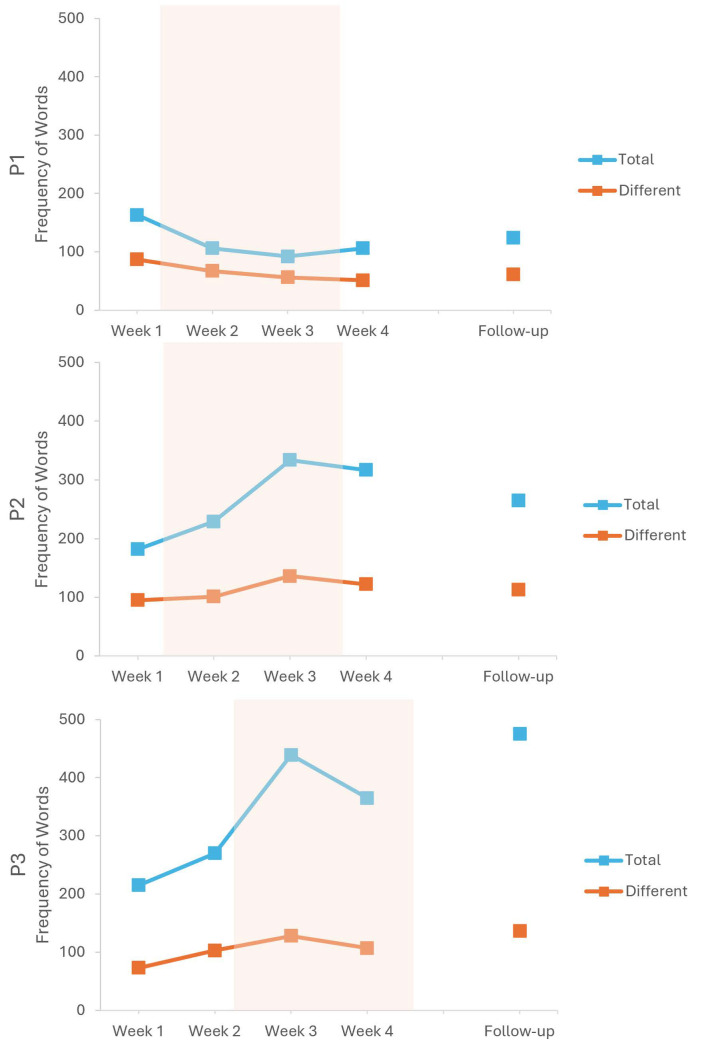
Frequency of words out of all words in each session during the PCR task. Lines show total number of words and number of different words. Shaded orange area indicates stimulation period.

Regarding the other measures, we found an increase in the mean length of utterance after the stimulation period for P2, which was not sustained at follow-up (see Figure A1). Other linguistic measures, such as fluency and word frequency, were not associated with the stimulation (see Figure A1 and Figure A2, and Table A4 and Table A5).

### 3.4. SAQOL-39

For P1, we observed an increase in the Communication and Physical subscores (Figure 6). A decrease was noted at Week 3 for both Psychological and Energy subscores, while these remained stable during the other weeks. At follow-up, only the Physical subscore remained higher compared to pre-intervention level. Qualitatively, P1 reported greater ease in communication within professional settings, particularly in group contexts.

For P2, no clear trends were observed across subscores, except for a small decrease in the Energy subscore in Week 3. Qualitatively, P2 reported improvements in communication with his young children.

For P3, the Communication subscore increased steadily from the first week of stimulation to the end of the intervention, although this improvement was not maintained at follow-up. Additionally, P3 reported improvements in reading, which were not formally assessed by the measures included in this study.

## 4. Discussion

This study aimed to improve lexicosemantic impairments in three persons with post-stroke aphasia using inhibitory rTMS targeting the right ATL. The findings can be summarized as follows: (1) in the Picture Naming task, all participants improved in accuracy (P1 and P3) and/or RT (P1 and P2), while only P1 showed improvement in accuracy for the Auditory Naming task; (2) in the Semantic Decision task, P2 showed improvement in both accuracy and RT, while P1 showed improvement in RT alone and P3 showed no improvement; (3) the PCR task showed a clear improvement for P3 alone; and (4) P1 and P3 reported changes in daily life communication that were not sustained at follow-up.

### 4.1. Effects on Naming

All participants showed improvement on at least one naming measure, most consistently for the Picture Naming task. Since rTMS induces effects not only on the targeted area but also on connected networks, we hypothesize that language improvement could be associated with the modulation of the ventral pathway via ATL stimulation, leading to specific improvement in lexical retrieval processes [10,14]. These results are consistent with several studies implicating ATL in tasks with a lexical component [16,19]. For example, a lesion-symptom mapping study of picture naming [41] identified the left ATL as critical but not the left IFG. Another study in neurotypical population using inhibitory rTMS targeting the left ATL showed specific impairments in picture naming RT [42]. The present study did not directly target the left ATL but instead applied inhibitory stimulation to the right ATL, following the principle of interhemispheric imbalance [7,8]. While our behavioral results are consistent with this theory, further work is needed to test whether the benefits are indeed due to suppression of an overactive Right Hemisphere, or due to a different mechanism, as some discrepancies have been noted in the literature. For instance, Lin et al. [43] used inhibitory rTMS targeting the pars triangularis of the right Inferior Frontal Gyrus in persons with post-stroke aphasia and reported language improvements associated with both decreased and increased right hemisphere connectivity, as measured by resting state fMRI. We also note that facilitatory stimulation of the left ATL may yield to similar effects, and need further exploration. Another point to consider is the effect of inhibitory vs. facilitatory rTMS on brain connectivity, while taking into account different types of aphasia, lesion profiles and residual network integrity [9].

### 4.2. Effects on Semantic Decision

The effect on the Semantic Decision task were more heterogeneous, with one participant showing no change after the intervention on this receptive task. Indeed, predicting rTMS efficacy, and non-invasive brain stimulation more broadly, in post-stroke aphasia is challenging. Several studies have highlighted the potential influence of aphasia type and lesion localization on treatment response [44,45]. Furthermore, the ATL is a key region involved in lexicosemantic processes, partly due to its extensive connectivity. First, both ATLs are interconnected. Moreover, superior, middle, and ventral ATL are connected to the angular gyrus, posterior MTG, IFG and medial prefrontal cortex functionally [46], and through several white matter tracts, including the uncinate, inferior longitudinal, middle longitudinal, and arcuate fasciculi [47]. These regions are central parts of the lexical semantic processing system [16,19]. Preservation of ATL connectivity may also predict the effectiveness of rTMS in different tasks, as stimulation can influence the entire connected network [10,14].

### 4.3. Generalization and Perspectives

The present study included participants with high SES scores, which occurred by chance. This unexpected factor may have influenced the results. First, our participants may have had higher expectations for language fluency and accuracy, making them more likely to present floor/ceiling effects, explaining some heterogeneous outcomes in the present study. Moreover, even mild deficit had a strong impact on quality of life for P1 and P2, and they both did not return to work after their stroke. When comparing with a similar high SES neurotypical population for the PCR task, we found improvement for P3 only. High SES may itself be a positive factor in rehabilitation outcomes [48], although this requires further investigation. Finally, P1 had moderate aphasia, whereas P2 and P3 had mild deficits. Notably, P1 showed more homogeneous and stronger naming improvements, possibly due to greater room for improvement, and aligning with previous studies showing that aphasia severity might influence response to rehabilitation depending on frequency and intensity [4].

In conjunction with a previous study focusing on motor cortex stimulation in people with post-stroke aphasia suffering from phonological impairments [13], the present study highlights the possibility of selecting cortical targets for rTMS based on a participant’s clinical profile. In the present study, the ATL was selected for participants with lexicosemantic impairments. However, given the small sample size, heterogeneity of effects, and absence of sham control, the present findings should be interpreted as preliminary. This approach could make rTMS more tailored to the patient’s profile or therapeutic goals, but it requires investigation in larger, sham-controlled clinical trials [13].

### 4.4. Combined Therapy and Long-Lasting Effects

The present study reported improvements for all participants after rTMS was administered as a standalone intervention, in line with previous findings in the literature [49,50,51]. However, several gains were not sustained at follow-up, which contrasts with most findings on rTMS in chronic post-stroke aphasia [1]. This may be attributable to the fact that rTMS was administered without concurrent SLT. Although the linguistic tasks were administered without corrective feedback and therefore do not constitute structured SLT, repeated task exposure may still influence performance. Future studies may therefore examine whether combining rTMS with targeted SLT enhances the long-lasting effect of improvements.

### 4.5. Limitations

One important limitation of this study concerns the calculation of the RMT. One participant’s hair affected the RMT measurement by increasing the distance to the target site. This issue is particularly relevant for ATL stimulation, since RMT is measured in regard to the motor cortex (closer to the top of the head) where hair is usually thicker, while the ATL target is typically on the temple (where hair is thinner or absent). This led to variations in RMT estimates and applied strength. The Sol Braiding Method might have provided more accurate RMT measure [52]. Furthermore, as this is the case for all rTMS study using RMT as a measure of cortical excitability when not targeting the motor cortex, the reliability of this measure at other brain regions is questionable and has been subject to debate [53]. Another limitation is that several participants showed a floor effect for RT in the Auditory Naming task. This was mainly due to the inclusion of participants with mild aphasia, making improvements harder to detect, and/or the fact that all the participants had a high socioeconomic status, with potentially higher premorbid linguistic ability. In addition, one participant began responding before the end of the stimuli during the rTMS intervention period. While this cannot be attributed to the intervention or repetition effects (see Results Section 3.1.2), it may have introduced differences compared with P1 and P3, who likely waited for the end of the description before answering. This issue should be considered when using an Auditory Naming task in an SCED model, where the same items are presented multiple times. Moreover, we only investigated long-term effects one month after the intervention, whereas some studies have shown longer-term effects of rTMS [5]. Finally, as is inherent to the SCED model, each participant is compared to themselves and there is no sham control. Future studies with a randomized sham-controlled trial to rule out a placebo effect would be informative.

## 5. Conclusions

This study highlighted the potential of targeting the right ATL with inhibitory rTMS in the recovery of post-stroke aphasia. More precisely, we hypothesized that stimulating the ATL modulates interconnected networks, particularly the ventral semantic pathway, leading to improvements in lexicosemantic processes. Each participant demonstrated improvement in at least one lexicosemantic measure after rTMS was applied without concurrent SLT. Given the small sample size and absence of sham control, these findings should be considered preliminary. This approach suggests a potential direction for future rTMS target selection based on each person with aphasia’s specific impairments and rehabilitation goals. Larger sham-controlled trials are needed to determine whether ATL stimulation can reliably support lexicosemantic recovery in post-stroke aphasia, in combination with SLT.

## Figures and Tables

**Table 1:** Demographic data of the participants and stroke information.

Participant	Gender	Age at Inclusion	Highest Level of Education Completed	Last or Current Occupation	SES ^1^ Scores (Hollingshead)	Time Post-Onset (Months)	Type of Stroke
P1	M	68	MD (Doctor of Medicine)	Ophthalmologist (last)	66	36	Ischemic
P2	M	45	PhD	Professor of history (last)	66	68	Ischemic
P3	W	44	JD (Juris Doctor)	Lawyer (current)	66	52	Ischemic

^1^ SES: Socioeconomic status.

**Table 2:** Tau statistical analyses for the Auditory and Picture Naming tasks, and Semantic Decision task. Scores in bold are statistically significant.

Task	Measure	Effect Size		
		P1	P2	P3
Picture Naming	Accuracy	**Tau = 0.703** ***p*** ** = 0.001, SE_Tau_ = 0.225**	Tau = 0.346 *p* = 0.099, SE_Tau_ = 0.297	**Tau = 0.569** ***p*** ** = 0.006, SE_Tau_ = 0.260**
	RT	**Tau = −0.665** ***p*** ** = 0.001, SE_Tau_ = 0.236**	**Tau = −0.578** ***p*** ** = 0.003, SE_Tau_ = 0.258**	Tau = −0.355 *p* = 0.070, SE_Tau_ = 0.296
Auditory Naming	Accuracy	**Tau = 0.459** ***p*** ** = 0.031, SE_Tau_ = 0.288**	Tau = 0.189 *p* = 0.387, SE_Tau_ = 0.311	Tau = 0.380 *p* = 0.076, SE_Tau_ = 0.292
	RT	**BCTau = 0.640** ***p*** ** = 0.001, SE_Tau_ = 0.249**	BC Tau = 0.008 *p* = 1.000, SE_Tau_ = 0.316	**BC Tau = 0.711** ***p*** ** = 0.000, SE_Tau_ = 0.222**
Semantic Decision	Accuracy	Tau = 0.387 *p* = 0.057, SE_Tau_ = 0.299	**Tau = 0.399** ***p*** ** = 0.044, SE_Tau_ = 0.290**	Tau = −0.162 *p* = 0.484, SE_Tau_ = 0.329
	RT	**Tau = −0.494** ***p*** ** = 0.014, SE_Tau_ = 0.282**	**Tau = −0.628** ***p*** ** = 0.001, SE_Tau_ = 0.246**	**BC Tau = 0.712** ***p*** ** = 0.001, SE_Tau_ = 0.234**

BC: Baseline-corrected; SE: standard error.

## Data Availability

The raw data supporting the conclusions of this article will be made available by the authors on request.

## Abbreviations

The following abbreviations are used in this manuscript:

ATL	Anterior Temporal Lobe
fMRI	Functional Magnetic Resonance Imaging
IFG	Inferior Frontal Gyrus
MTG	Middle Temporal Gyrus
PCR	Peer Conflict Resolution
RMT	Resting Motor Threshold
RT	Reaction Time
rTMS	Repetitive Transcranial Magnetic Stimulation
SAQOL-39	Stroke and Aphasia Quality of Life Scale—39 items
SCED	Single-Case Experimental Design
SES	Socioeconomic Status
SLT	Speech and Language Therapy
tDCS	Transcranial Direct Current Stimulation
WAB	Western Aphasia Battery

## Appendix A

**Table A1:** Language score for the WAB and the Philadelphia Naming Test.

Subtest	P1	P2	P3
Bedside Language Score (WAB) (max = 100, sum of scores ÷ 8 × 10)	88.75	92	91.25
Spontaneous Speech: Content (max = 10)	8	10	10
Spontaneous Speech: Fluency (max = 10)	8	8	8
Auditory Verbal Comprehension (max = 10)	9	9	10
Sequential Commands (max = 10)	10	8	10
Repetition (max = 10)	9	7	7
Object Naming (max = 10)	9	10	8
Reading (max = 10)	10	10	10
Writing (max = 10)	8	10	10
Philadelphia Naming Test 30	28	30	30

**Table A2:** Mean values and standard deviations of variables for Picture and Auditory Naming tasks in Original and Supplementary items.

	Picture Naming		Auditory Naming	
	Original	Supplementary	Original	Supplementary
Age of Acquisition	4.93 (0.94)	10.12 (1.12)	5.98 (1.23)	7.27 (2.98)
Concreteness	4.88 (0.13)	4.87 (0.10)	4.77 (0.23)	2.17 (0.45)
Frequency	2.55 (0.56)	2.09 (0.34)	2.54 (0.48)	3.62 (0.88)

**Table A3:** Examples of rating justifications for the semantic coherence of the PCR task, generated by CHATGPT-5 for P1.

	Retelling	Questions
Participant 1 week 1	Score = 40 The story is severely compressed and inaccurate. Roles are reversed (Mike asks Peter to switch). Key elements are missing (grill vs. garbage, sore arm). Narrative lacks sequencing or causal structure.	Score = 50 Identifies a general issue (“not working as a team”) but not the specific conflict. Reasoning is abstract and detached from the story. Introduces extreme outcomes (“violence”) without logical connection. Some attempt at explanation, but poorly grounded.
Participant 1 week 2	Score = 38 Very vague and hesitant (“I think that’s what’s that”). Reversal of who asks to switch jobs. Almost no descriptive detail or structure.	Score = 46 Emphasizes communication in general terms. Responses trail off and lose coherence. Logical connections are weak and incomplete.
Participant 1 week 3	Score = 44 Corrects character names after hesitation. Basic conflict is present (request to switch, refusal). Still minimal detail, but clearer than earlier sessions.	Score = 52 Identifies refusal to switch as the problem. Reasoning is simplistic but logically consistent. Offers compromise as a solution without elaboration.
Participant 1 week 4	Score = 42 Confusion about who wants to switch jobs persists. Story structure remains incomplete. Conflict is stated but not clearly framed.	Score = 50 Recognizes negotiation as a solution. Cause–effect logic is present but shallow (“that would solve the problem”). Emotional outcomes are minimally addressed.
Participant 1 follow-up	Score = 43 Slightly clearer sequencing. Still repetitive and lacking essential story details.	Score = 51 Identifies refusal as the core issue. Predicts emotional outcomes with basic logic. Reasoning remains generic and under-specified.
Participant 2 week 1	Score = 62 Core elements are present (grill, trash, sore arm). Sequence is mostly correct. Some disfluencies and name uncertainty	Score = 68 Clearly identifies the conflict and motivations. Uses logical reasoning (entitlement, fairness). Some speculation, but reasoning remains coherent.
Participant 2 week 2	Score = 64 Story elements are clearly identified. Logical sequence maintained. Slight redundancy but coherent.	Score = 70 Demonstrates boundary-setting reasoning. Clear cause–effect explanations. Occasional irrelevant speculation (personality traits).
Participant 2 week 3	Score = 66 Structured retelling with clear roles and tasks. Logical progression from assignment to conflict.	Score = 74 Multiple solution pathways outlined. Strong causal reasoning and clear justification. Minor role-play digressions.
Participant 2 week 4	Score = 65 Story is coherent but hesitant. Some uncertainty about outcomes.	Score = 71 Clear logical stance and conflict resolution. Reflects on uncertainty without contradiction.
Participant 2 follow-up	Score = 66 Accurate, well-sequenced retelling. No major inconsistencies.	Score = 72 Manager-based resolution is logically justified. Cause–effect reasoning is explicit.
Participant 3 week 1	Score = 57 Story structure present but hesitant and repetitive. Confusion in phrasing and task description.	Score = 63 Identifies conflict accurately. Reasoning incomplete but logically aligned with story.
Participant 3 week 2	Score = 60 Clearer and more concise. Maintains correct sequence.	Score = 66 Proposes solutions logically. Considers consequences with minimal contradiction.
Participant 3 week 3	Score = 67 Complete, accurate retelling. Strong narrative structure.	Score = 73 Explores multiple solutions logically. Clear cause–effect relationships.
Participant 3 week 4	Score = 69 Confident, well-structured retelling. No inconsistencies.	Score = 75 Logical, balanced reasoning. Strong justification for chosen solutions.
Participant 3 follow-up	Score = 71 Fully accurate, fluent, and well organized.	Score = 77 Clear problem definition. Multiple coherent solutions with closure. Explicit emotional and practical outcomes.

**Table A4:** Fluency (P1, P2 and P3) in raw counts and percentages during the PCR task. Percentage is error over intended words in the sample. Typical disfluencies include phonological fragments, phrase revisions, pauses, and filled pauses. Stimulation period include Weeks 2–3 for P1 and P2, Weeks 3–4 for P3.

	**Phonological Fragments**	**Phrase Revisions**	**Pauses**	**Filled Pauses**	**Typical Disfluencies**	**Total Disfluencies**	**% Total Disfluencies**
P1							
Week 1	0	0	19	16	35	35	23.19
Week 2	0	1	10	0	11	11	9.09
Week 3	0	0	12	0	12	12	9.45
Week 4	0	0	12	0	12	12	9.45
Follow-up	0	0	9	0	9	9	5.81
P2							
Week 1	0	3	0	26	29	29	12.89
Week 2	0	1	5	27	33	33	11.54
Week 3	2	2	4	20	28	29	6.87
Week 4	3	0	16	36	55	55	13.72
Follow-up	0	0	2	0	2	2	0.60
P3							
Week 1	0	0	0	0	0	0	0
Week 2	0	0	15	0	15	16	4.73
Week 3	0	0	9	0	9	9	2.08
Week 4	0	0	9	0	9	9	2.08
Follow-up	0	0	1	0	1	1	0.17

**Table A5:** Total number of words, number of different words, and Type/Token ratio (TTR) during the PCR task. “Tokens” are total number of items (words), and the ratio is a measure of lexical diversity. Number ranges from 0.001 to 1.0. A low value indicates a lot of repetition and a high value means each word in the sample was different. Stimulation period include Weeks 2–3 for P1 and P2, Weeks 3–4 for P3.

	Different Words	Total Words	TTR
P1			
Week 1	87	163	0.534
Week 2	67	106	0.632
Week 3	56	92	0.609
Week 4	51	106	0.481
Follow-up	61	124	0.492
P2			
Week 1	95	182	0.522
Week 2	101	229	0.441
Week 3	136	334	0.407
Week 4	122	317	0.385
Follow-up	113	265	0.426
P3			
Week 1	73	215	0.340
Week 2	103	270	0.381
Week 3	128	439	0.292
Week 4	107	365	0.293
Follow-up	136	475	0.286
